# Preoperative Stereotactic Radiosurgery of Brain Metastases: Preliminary Results

**DOI:** 10.7759/cureus.1987

**Published:** 2017-12-26

**Authors:** Elena Vetlova, Denis A Golbin, Andrey V. Golanov, Alexander A Potapov, Sergey M Banov, Natalia Antipina, Valery V Kostjuchenko, Dmitry Y Usachev, Artem Y Belyaev, Sergey Goryaynov

**Affiliations:** 1 Department of Radiation Oncology, N.N. Burdenko National Medical Research Center for Neurosurgery; 2 Skull Base and Craniofacial Surgery, N.N. Burdenko National Medical Research Center for Neurosurgery; 3 Director, N.N. Burdenko National Medical Research Center for Neurosurgery; 4 Nuclear Medicine Department, N.N. Burdenko National Medical Research Center for Neurosurgery; 5 Gamma Knife, Neurosurgery Business Center; 6 Vice-Director, Department of Vascular Neurosurgery, N.N. Burdenko National Medical Research Center for Neurosurgery; 7 Department of Vascular Neurosurgery, N.N. Burdenko National Medical Research Center for Neurosurgery; 8 Department of Neurotrauma, N.N. Burdenko National Medical Research Center for Neurosurgery

**Keywords:** brain metastasis, preoperative stereotactic radiosurgery, combined treatment, surgical resection, up-front treatment

## Abstract

Introduction: Preoperative stereotactic radiosurgery (pre-SRS) is a recent advancement in the strategy for brain metastasis (BM) management, and available data demonstrate the advantages of pre-SRS before postoperative radiation treatment, including lower rates of local toxicity, leptomeningeal progression, and a high percentage of local control. The authors presented the results of pre-SRS in patients with BM.

Materials and methods: Nineteen patients with BM (11 female and eight male) have been treated at N.N. Burdenko Medical Research Center for Neurosurgery (Moscow, Russia) and Gamma-Knife Center (Moscow, Russia) using pre-SRS. A total of 22 symptomatic metastatic lesions were preoperatively irradiated in the series. Eight patients had multiple BM (number of metastases ranged between two and seven). The median target volume for combined treatment was 14.131 cc (volumes varied between 2.995 and 57.098 cc; mean - 19.986 cc). The median of the mean target dose was 18 Gy, ranging between 12.58 and 24.36 Gy.

Results: All patients tolerated pre-SRS well, without any neurological deterioration, and surgical treatment was performed as scheduled. The median follow-up period was 6.3 months (ranging between five weeks and 22.9 months). In 17 out of 19 patients, follow-up magnetic resonance (MR) images obtained two or three months after the combined treatment demonstrated the postoperative cavity without any signs of postradiation alterations in the perifocal tissues. In two observations, peritumoral edema was present. Local recurrences were found in two cases, 5.5 and 17.4 months after treatment. Radionecrosis was present in one observation after 4.6 months of follow-up. Two patients died of disease progression and are presented as illustrative cases.

Conclusion: The combined treatment of secondary brain tumors has proved to be the best treatment option. Preoperative stereotactic radiosurgery may decrease radiation-induced toxicity and rates of local tumor progression. The potential hazards of pre-SRS associated with the postoperative healing of irradiated soft tissues of the head were not confirmed in our study. The decision of pre-SRS should be made by the tumor board, including specialists in neurosurgery, neuro-oncology, and radiation oncology, if the diagnosis of BM is based on oncological history and visualization data.

## Introduction

Current guidelines for the treatment of patients with brain metastasis (BM) evolved during recent decades. Advances in surgical techniques, neurovisualization, radiation, and systemic therapy resulted in increased overall survival in this cohort of patients.

It was shown that surgical treatment of BM may lead to longer survival of the patients and a better functional outcome of treatment [1]. At the same time, a combination of surgery with radiotherapy is essential to achieve local control of BM after surgical resection. Whole brain radiation therapy (WBRT) allowed decreasing of the local recurrence rate to 28% at one year after treatment, however, it was proved to cause significant neurotoxicity and was replaced by postoperative stereotactic radiosurgery (SRS) and hypofractionated radiotherapy. The latter is associated with less frequency of radionecrosis in comparison with radiosurgery [2].

Preoperative stereotactic radiosurgery (pre-SRS) is a recent advancement in the strategy of BM management, and available data demonstrate the advantages of pre-SRS before postoperative radiation treatment, including lower rates of local toxicity, leptomeningeal progression, and a high percentage of growth control [3-4].

In the given study, we present the results of pre-SRS in patients with BM, with selected case descriptions.

## Materials and methods

Since August 2015, 19 patients with BM (11 female and eight male) have been treated in N.N. Burdenko Medical Research Center for Neurosurgery (Moscow, Russia) and Gamma-Knife Center (Moscow, Russia) using pre-SRS. The characteristics of the series are summarized in Table 1.

**Table 1:** Characteristics of the patients (summary)

Parameter		Patients (Lesions)
Gender		
	Male	8
	Female	11
Median of age (years)		56 (range 30-71)
Histological diagnosis of the primary tumor		
	Breast cancer	4
	Kidney cancer	4
	Melanoma	4
	Non-small cell lung cancer	4
	Colorectal cancer	2
	Cervical cancer	1
Properties of the resected BM		
	Primary	11 (14)
	Local recurrence after stereotactic radiotherapy or surgery	8 (9)
RPA		
	1	5
	2	9
	3	5
Extracranial progression of the disease		
	Yes	8
	No	8
	Data not available	3
Karnofsky performance score		
	< 80	5
	80 and higher	14
Localization		
	Supratentorial	14 (16)
	Infratentorial	4 (4)
	Both	1 (2)
Number of BM		
	Single BM	16 (16)
	Multiple BM	3 (6)
Median lesion volume (cc)	14.560 (range 2.995 – 57.098)	
Меdian of mean dose (Gy)	18.05 (range 12.58 – 24.36)	

A total of 22 symptomatic metastatic lesions were preoperatively irradiated in the series. Three patients had two metastases; in all of them, the lesions were irradiated n a single procedure. Eight patients had multiple BMs (number of metastases ranged between two and seven). They underwent radical radiosurgical treatment of concurrent BMs.

The median target volume for combined treatment was 14.131 cc (volumes varied between 2.995 and 57.098 cc; mean - 19.986); see Table 2. Eight patients in this study had local BM recurrences after the previous treatment. In five of them, the tumor was complicated by radionecrosis after previous stereotactic radiotherapy, and three had recurrences after only surgical BM treatment. The median of the mean target dose was 18 Gy, ranging between 12.58 and 24.36 Gy. The treatment was carried out using 6 MeV Novalis linear accelerators (Brainlab, Munich, Germany), CyberKnife (Accuray, Sunnyvale, California, United States), and Gamma Knife (Elekta, Stockholm, Sweden). The surgical part of the combined treatment was performed within 24-48 hours after radiosurgery.

**Table 2:** Pre- and post-treatment characteristics of the patients The last five columns refer to the results. EC - extracranial, KPS – Karnofsky performance score, NSCLC – non-small cell lung cancer, RPA – recursion partitioning analysis

#	Age	Primary diagnosis	KPS	RPA	Extracranial metastases	Mean pre-SRS dose, Gy	No of lesions (pre-SRS)	Volume of treated lesions (pre-SRS), cm^3^	Resected lesion	Edema	Radionecrosis	Progression	Follow-up, months	Status
1	49	melanoma	70	3	stable disease	18.53	1	12.087	primary	no	no	distant recurrence	11.3	death
2	60	breast cancer	80	1	no EC disease	20.13	1	8.706	recurrent	yes	yes	local recurrence	22.9	alive
3	57	kidney cancer	80	2	progression	24.36	1	4.912	primary	no	no	distant recurrence	10.0	alive
4	57	breast cancer	80	2	N/A	17.04/ 17.45	2	25.550/ 14.627	primary	no	no	distant recurrence	8.9	alive
5	55	breast cancer	80	2	progression	18.00	1	24.850	recurrent	yes	yes	no	7.8	death
6	30	NSCLC	80	2	N/A	17.00	1	14.560	recurrent (no irradiation)	no	no	distant recurrence	7.1	alive
7	56	kidney cancer	70	3	progression	24.00/ 17.91	2	32.820/ 12.500	primary	no	no	no	2.4	alive
8	42	melanoma	90	1	no EC disease	19.94	1	13.192	recurrent	no	no	no	10.0	alive
9	52	melanoma	80	2	progression	18.00	1	22.297	primary	yes	no	local recurrence	17.9	alive
10	51	NSCLC	80	2	N/A	18.00	1	13.086	primary	no	no	no	6.3	alive
11	39	melanoma	80	1	no EC disease	19.86/ 20.17	2	13.406/ 23.749	primary	yes	no	local leptomeningeal disease	6.7	alive
12	64	cervical cancer	70	3	stable disease	14.80	1	40.800	primary	yes	no	no	3.2	alive
13	48	breast cancer	80	1	no EC disease	23.70	1	2.995	recurrent	yes	no	no	5.1	alive
14	58	NSCLC	70	3	no EC disease	20.00	1	10.198	recurrent (no irradiation)	no	no	no	2.4	alive
15	71	kidney cancer	80	1	no EC disease	18.10/ 12.58	1	9.507/ 22.940	recurrent (no irradiation)	no	no	no	3.2	alive
16	41	colorectal cancer	80	2	progression	17.54	1	18.263	recurrent	no	no	no	3.1	alive
17	56	kidney cancer	70	3	progression	17.90	2	32.870	primary	no	no	no	2.1	alive
18	51	NSCLC	90	2	progression	22.00	1	8.414	primary	no	no	no	1.7	alive
19	61	colorectal cancer	80	2	progression	16.95	1	57.098	primary	no	no	no	1.3	alive

## Results

All patients tolerated pre-SRS well, without any neurological deterioration, and surgical treatment was performed as scheduled. The median follow-up period was 6.3 months (ranging between five weeks and 22.9 months). In 17 out of 19 patients, follow-up magnetic resonance (MR) images obtained two or three months after the combined treatment demonstrated a postoperative cavity without any signs of postradiation alterations in the perifocal tissues. In two observations, peritumoral edema was present.

Local recurrences were found in two cases, 5.5 and 17.4 months after treatment. Local leptomeningeal disease was discovered 1.5 months after partial resection of BM proximal to the dura in a single case. Radionecrosis was present in one observation after 4.6 months of follow-up (pre-SRS was secondary radiation therapy in this patient, and radionecrosis coincided with tumor recurrence before the next surgical treatment).

To summarize the pre- and post-treatment data and outcomes, the details are shown in Table 2. Two patients died of disease progression; both cases are presented below.

Illustrative case 1

A 50-year-old female with a history of known melanoma complained of fatigue and weakness in the legs in May 2015. An MR study discovered a cystic, solid tumor in the left frontal lobe with contrast enhancement and peritumoral edema. The diameter of the lesion was 3.5 cm. The other tumor was found in the right frontobasal area. Its diameter was 5 mm. Steroid therapy resulted in partial regression of the symptoms.

The patient had been under medical surveillance due to dorsal skin melanoma T3N2cM0 since 2012. She underwent surgical treatment and interferon immunotherapy. A histological analysis demonstrated a nodular type of melanoma without ulceration. The Breslau grade was 3 mm infiltration. There were small satellites in the dermis, BRAF-positive.

The disease progressed after one year: metastatic lesions were found in the liver, spleen, and axillary lymphatic nodes. Three cycles of chemotherapy were performed using the following regimen: dacarbazine (800 mg/m^2^ on the first day of the cycle), cisplatin (20 mg/m^2^ during the first five days of the cycle), followed by ipilimumab (3 mg/kg, a total of four injections per cycle). The treatment resulted in a partial regression of the extracranial metastases. Ten months later (May 2015), a brain MR showed three BMs up to 5 mm in diameter, with foci in the occipital lobe, in the frontal lobe, and in the parietooccipital region of the right hemisphere.

The patient underwent CyberKnife stereotactic radiosurgery on June 1, 2015. A total of 24 Gy were delivered to the metastases in the frontal, parietooccipital, and occipital regions of the right hemisphere. The volume of the foci was 0.936, 0.652, and 0.161 cc, respectively, with a median dose of 22 Gy prescribed to 79% isodose.

Follow-up MR images in August 2015 showed signs of progression of the irradiated foci, which were estimated as post-radiation alteration according to the computed tomography (CT) perfusion study, as well as new lesions in the right (5 mm) and left (35 mm) frontal lobes. A CT-perfusion study verified active local recurrences.

Combined treatment was undertaken, including pre-SRS with the surgical removal of the BM from the left frontal lobe.

**Figure 1:**
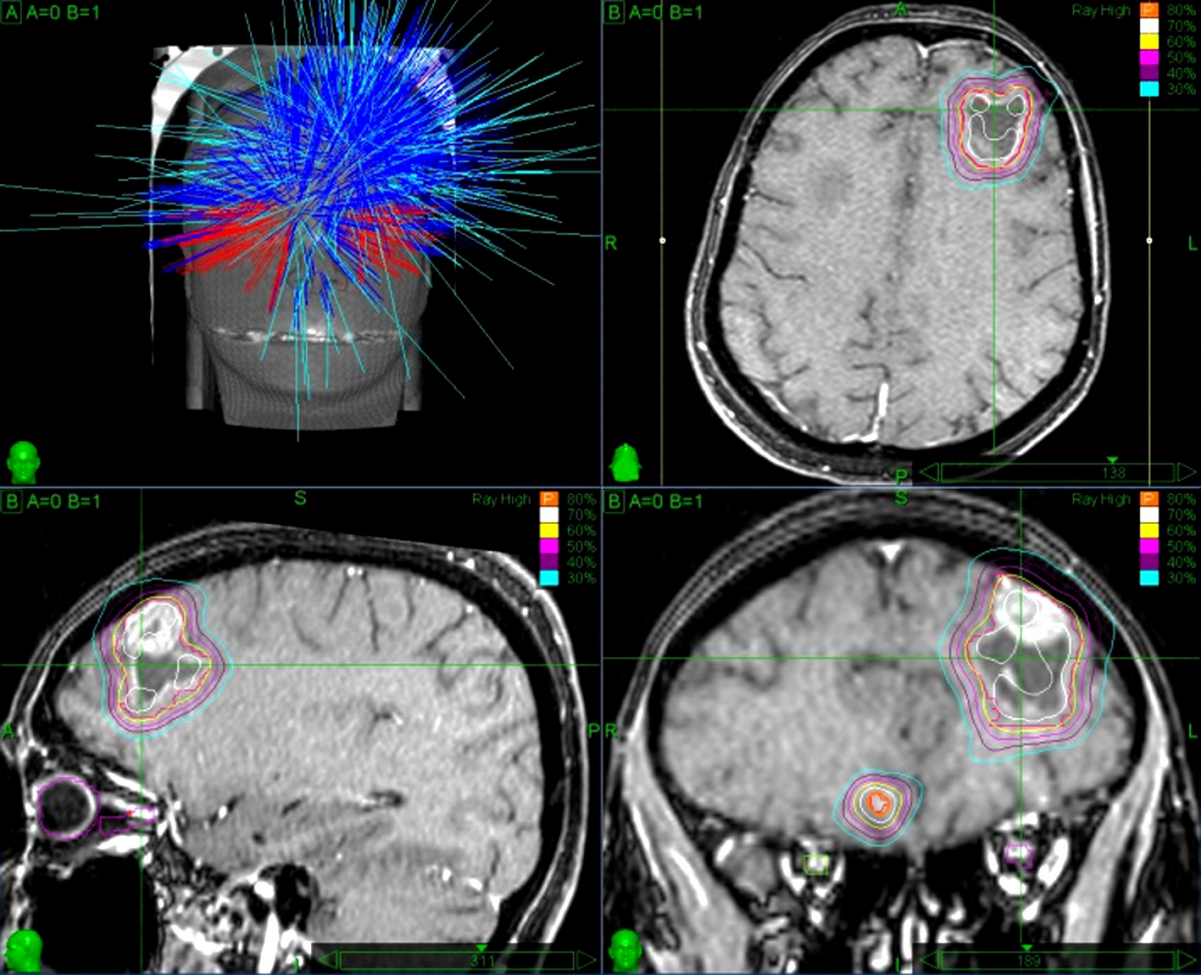
Irradiation plan of pre-SRS of patient 1 pre-SRS: preoperative stereotactic radiosurgery

The patient underwent CyberKnife radiosurgical treatment of the right frontal lobe lesion as the single procedure and neoadjuvant treatment of the left frontal lobe lesion on August 26, 2015. Target volumes comprised 0.248 cc and 12.087 cc. The median delivered doses were 24.08 Gy and 18.53 Gy, respectively. Prescription to the 80% isodose line was 21.8 Gy (Figure 1).

The microsurgical resection of the melanoma metastasis from the left frontal lobe using ultrasonic guidance was accomplished on August 27, 2015. The biopsy report showed metastasis of the melanoma with necrobiotic changes in the endothelium and vascular walls (CD31, CD34). Postoperative course and wound healing were uneventful. By the time of discharge, the patient was in good condition without any noticeable neurological deficit. Early postoperative CT scans showed no residual tumor or complications.

Twelve months later, it was found that the patient was treated with vemurafenib 960 mg bid during one year. According to examination reports, pulmonary foci regressed; however, the metastatic lesion in the hepatic hilus enlarged.

**Figure 2:**
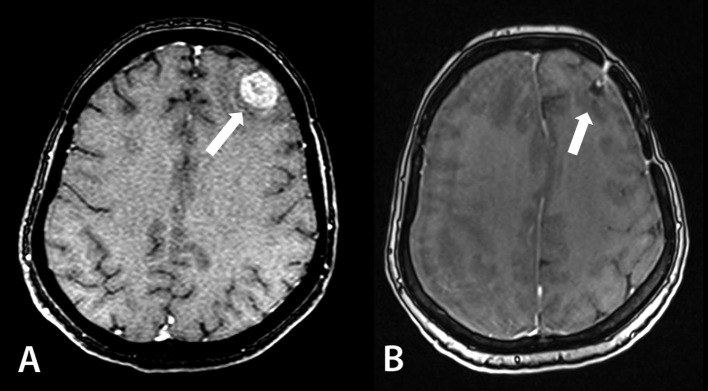
MR images of patient 1 before combined treatment (A) and after 12 months of follow-up (B). No radiation-induced toxicity in the metastatic tumor bed in the left frontal lobe is noticed. MR: magnetic resonance

Follow-up MR images of the brain (July 2016) demonstrated an enlargement of the irradiated metastases in the right occipital and parietooccipital regions due to intratumoral hemorrhage; slight enlargement of cyst in the right frontal lobe; and progression of the peritumoral edema with mass effect and brain dislocation. A combined treatment of the focus in the left frontal lobe resulted in complete regression without any signs of radiation-induced toxicity (Figure 2). Two new BMs were found in the brain.

One month later, the patient died of bleeding from esophageal veins in the setting of the progression of the liver metastases.

Illustrative case 2

A 56-year-old female patient presented with severe headaches responsive to dexamethasone and generalized seizures in October 2016. Her MR images showed a tumor in the right parietooccipital region with a diameter up to 36 mm, with a dural attachment and significant peritumoral edema.

In 2010, she underwent combined treatment for breast cancer T2N0M0 (mastectomy and adjuvant radiotherapy). The histological diagnosis was a lobular invasive carcinoma, without lymphatic node involvement, triple-negative phenotype.

In March 2014, metastatic foci were found in the lungs and mediastinal lymphatic nodes. After six cycles of chemotherapy, these lesions partially regressed.

On October 24, 2014, the patient developed a single seizure episode. CT and MR scans showed a BM in the right parietal lobe (dimensions 16 x 21 x 17 mm) with peritumoral edema.

Hypofractionated stereotactic CyberKnife radiotherapy of the BM was performed from November 27 – December 3, 2014. The target volume was 4.666 cc. The mean daily dose was 6 Gy and the total dose was delivered in five fractions. Thus, the mean delivered dose was 26.7 Gy prescribed to the 80% isodose line.

During seven months, she received capecitabine monotherapy.

**Figure 3:**
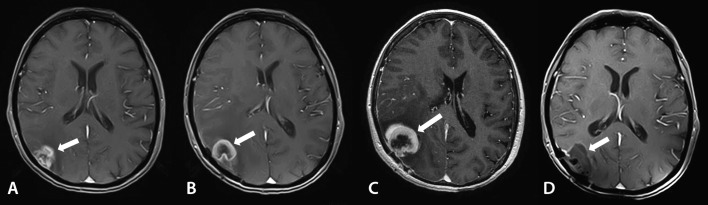
Patient 2: Left to right: A: first recurrence 11 months after radiotherapy; B: second recurrence after 2.5 months; C: progression of the recurrent tumor four weeks before combined treatment; D: complete regression without signs of radiation-induced toxicity after 2.5 months

In October 2015, the lesion in the right parietal lobe enlarged (Figure 3A). A CT-perfusion study confirmed tumor progression along with radionecrosis. Peritumoral edema also became more prominent. No new foci were found.

The BM in the right parietooccipital region was completely removed using ultrasound guidance on October 30, 2015.

Two months later, the extracranial progression of the disease was noticed. A lesion in the superior lobe of the right lung slightly enlarged, and a new epinephral metastatic focus was detected. A follow-up brain MR study showed the second recurrence of the resected BM without any new foci in January 2016. Images demonstrated a recurrent tumor in the right parietooccipital region up to 36 mm in diameter. Local peridural spread and severe peritumoral edema were present (Figure 3B).

Due to previous radiation therapy with consequent radionecrosis, and recurrences of the BM, a decision was made to perform combined treatment consisting of pre-SRS and reoperation.

**Figure 4:**
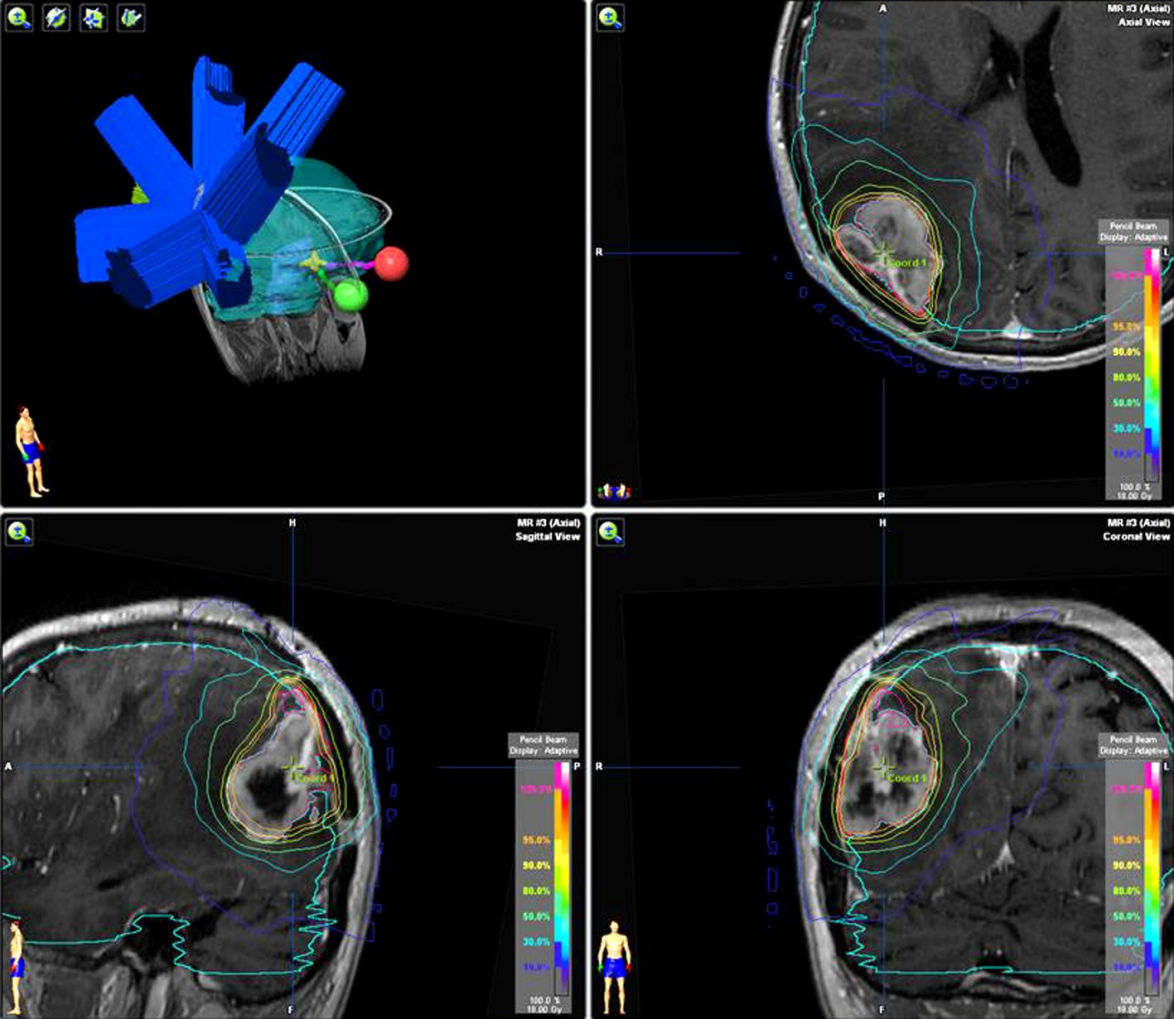
Plan of pre-SRS of patient 2 pre-SRS: preoperative stereotactic radiosurgery

On February 8, 2016, radiosurgery of the BM in the right parietooccipital region was performed using Novalis Linear Accelerator (Figure 3C). The dural attachment was also included in the treatment plan. The target volume was 24.85 cc. The median delivered dose was 18 Gy. Irradiation was accomplished from nine conformal fields with one isocenter (Figure 4). The next day, this metastatic lesion was resected. The histological report confirmed breast carcinoma metastasis with a significant necrotic component.

The postoperative course was uneventful. The patient was discharged in good condition with an absence of a noticeable neurological deficit. Postoperative CT scans with contrast enhancement did not show a visible residual tumor.

On the MR images obtained in April 2016, a postoperative cyst was present along with the complete regression of the solid tumor and the absence of postradiation changes. Perifocal edema was moderate. No new foci were detected (Figure 3D).

Due to the absence of prescribed systemic therapy, metastatic foci in both lungs progressed. During the next six months, the patient received capecitabine.

The appearance of headaches and dizziness resulted in the administration of steroids.

A follow-up MR study (June 2016) revealed focal contrast enhancement around the postoperative area, predominantly in the upper-lateral region, and increased peritumoral edema. A CT perfusion study confirmed postradiation alterations. New foci were not detected again.

On October 2, 2016 (eight months after combined treatment of the BM), the patient died of extra- and intracranial disease progression.

## Discussion

One of the critical trends in the evolution of treatment approaches is the strategy of combined management of BM. Patchell et al., in their fundamental study, demonstrated that surgical treatment may lead to an increase in the survival and long-term preservation of good functional status [1]. Postoperative radiotherapy is essential to achieve growth control of BM after surgical treatment. Postoperative WBRT allows a decrease in the local recurrence rate at one year after treatment from 46%-59% after surgical treatment to 28% [2].

However, due to the neurotoxic effects of WBRT, alternative treatment regimens should be found. The importance of finding the optimal type of radiation therapy is mainly based on the increased survival of oncologic patients. At the same time, the preservation of good quality of life is the second priority.

In this situation, postoperative radiosurgery of the metastatic tumor bed improves local growth control without WBRT, thus decreasing the risk of cognitive decline and preserving the level of quality of life [5].

Most of the data in the literature have shown that the local growth control rate after the postoperative radiosurgery of the metastatic tumor bed exceeds 70%, but the incidence of symptomatic radionecrosis is relatively high (17.5%). The high rates of radiation-induced complications are explained by the large target volume. On one hand, the dimensions of the tumor bed are large; on the other hand, the presence of micrometastases in the adjacent tissues leads to an increased area of irradiation [6-7].

One of the options for decreasing the risk of radiation-induced complications is a hypofractionated regimen of radiation therapy, which allows escalating the radiation dose, providing good local growth control, and lowering the incidence of radionecrosis [8].

Another management strategy that may minimize radiation-induced complications is radiosurgery followed by the surgical resection of a metastatic tumor within 24 hours. The available limited data of retrospective studies have shown the advantages of pre-SRS, including lower rates of local toxicity, leptomeningeal progression, and a high percentage of growth control comparable with adjuvant stereotactic radiotherapy. According to Johnson et al., at one year after SRS only (without surgical resection), the cumulative incidence of leptomeningeal disease, with death as a competing risk, was 5.2% versus 16.9% for patients treated with surgery and subsequent SRS (p < 0.01) [9].

Originally, the first results of pre-SRS were published by Asher et al. in 2014. They reported good local control of metastatic foci without documented radionecrosis after pre-SRS in 47 patients with BM. Local control, even after treatment of large tumors, comprised 97.8%, 85.6%, and 71.8% at six, 12, and 24 months of follow-up, respectively [3].

In 2016, Patel et al. (including Asher, one of the co-authors) published their results of the treatment of 66 patients who underwent pre-SRS. Local recurrences were observed in 24.5% of patients after two years, and leptomeningeal progression was present in only 3.5%. In this larger study with a longer follow-up period, the incidence of radionecroses did not exceed 5.6% [4]. Authors reduced the radiation dose by approximately 20% compared to standard dosing as per RTOG 90-05 [10]; however, in our study, the radiation dose was increased by 20% (see Table 2).

The planning of radiosurgery was significantly simplified in comparison with the postoperative treatment protocols due to the absence of vague boundaries of the postoperative cavity according to MR imaging (MRI) data. Most patients were treated using conformal planning. In addition, pre-SRS was associated with a shorter duration of treatment because the patient did not need radiotherapy from three to six weeks after the surgical resection of the BM. As a result, systemic therapy could be initiated immediately.

Pre-SRS is a new paradigm of combined treatment. It seems to be advantageous over the postoperative radiosurgery or hypofractionated radiotherapy of the tumor bed after surgery.

First, we suppose that the probability of a "good response" of the lesion to radiation treatment may be higher due to preserved vascularization and the absence of a postoperative hypoxic cavity.

Second, the process of delineation of an intact metastatic focus is significantly easier than the delineation of a postoperative cavity with unclear localization and volume of residual tumor according to MR data. Postoperative radiosurgery also cannot properly consider possible surgical seeding of the tumor. Postoperative irradiation requires the inclusion of an additional marginal zone, which is avoided in case of radiosurgery for an intact BM [11-13].

Third, since the tumor is subject to further complete or near-complete resection, the radiation dose may not be reduced according to the volume of the lesion. This may improve local control and help reduce the risk of radiation-induced toxicity.

Fourth, the seeding potential of irradiated cells is already significantly reduced, thus, a decrease in local recurrence rate and postoperative leptomeningeal dissemination may be obtained.

An additional advantage is the possibility of the simultaneous radical radiosurgical treatment of concurrent cerebral metastatic lesions that are not subject to surgical excision. Therefore, there is no need for the postoperative irradiation of these foci.

The relative disadvantages of pre-SRS include the possibility of a difference in the preoperative and postoperative diagnosis. Few publications emphasized this difference in 2%-11% of cases [1,14]. Two patients were not included in our study due to the difference in the preoperative and postoperative diagnoses. In one patient, lymphoma of the central nervous system was discovered; the other histological analysis revealed glioblastoma.

Limitations of the study

The main weaknesses of our study include small size and a relatively short follow-up period. Continuation of the treatment protocol and further follow-up surveillance is critical to formulate the final conclusions. Future studies should be done with a larger series size.

## Conclusions

The combined treatment of secondary brain tumors has proved to be the best treatment option. Preoperative stereotactic radiosurgery may decrease radiation-induced toxicity and rates of local tumor progression as well as leptomeningeal disease.

The potential hazards of pre-SRS associated with the postoperative healing of irradiated soft tissues of the head were not confirmed in our study.

The decision of pre-SRS should be made by the tumor board, including specialists in neurosurgery, neuro-oncology, and radiation oncology, if the diagnosis of BM is based on oncological history and visualization data.
